# tiRNAs: Insights into Their Biogenesis, Functions, and Future Applications in Livestock Research

**DOI:** 10.3390/ncrna8030037

**Published:** 2022-05-26

**Authors:** Fabio Sarais, Alvaro Perdomo-Sabogal, Klaus Wimmers, Siriluck Ponsuksili

**Affiliations:** 1 Institute for Genome Biology, Research Institute for Farm Animal Biology (FBN), Wilhelm-Stahl-Allee 2, 18196 Dummerstorf, Germany; sarais@fbn-dummerstorf.de (F.S.); perdomo-sabogal@fbn-dummerstorf.de (A.P.-S.); wimmers@fbn-dummerstorf.de (K.W.); 2 Faculty of Agricultural and Environmental Sciences, University Rostock, 18059 Rostock, Germany

**Keywords:** angiogenin, tRNA, cleavage, tiRNA, stress, immunity, tsRNAs, tRFs, sncRNA

## Abstract

Transfer RNA (tRNA)-derived small RNAs (tsRNAs) belong to a group of transfer ribonucleic acid (tRNA)-derived fragments that have recently gained interest as molecules with specific biological functions. Their involvement in the regulation of physiological processes and pathological phenotypes suggests molecular roles similar to those of miRNAs. tsRNA biogenesis under specific physiological conditions will offer new perspectives in understanding diseases, and may provide new sources for biological marker design to determine and monitor the health status of farm animals. In this review, we focus on the latest discoveries about tsRNAs and give special attention to molecules initially thought to be mainly associated with tRNA-derived stress-induced RNAs (tiRNAs). We present an outline of their biological functions, offer a collection of useful databases, and discuss future research perspectives and applications in livestock basic and applied research.

## 1. Introduction

Genome conservation of nucleotides located outside the exons of protein-coding genes increases with the complexity of the organisms across different phyla [1,2,3]. An organism’s complexity seems more related to the quantity of non-coding RNA genes (ncRNAs) than to the number of protein-coding genes [4].

Non-coding RNAs can be divided into two main groups depending on their transcription length: small ncRNAs (sncRNAs) and long ncRNAs (lncRNAs) [5,6]. Small ncRNAs are characterised by a diverse list of recently found RNA species, many of which are linked to the 5′ or 3′ regions of protein-coding genes. This class comprises micro RNAs (miRNAs), small interfering RNAs (siRNAs), piwi-interacting RNAs (piRNAs), small nuclear RNA (snRNAs), small nucleolar RNA (snoRNAs), centromere repeat-associated small interacting RNAs (crasiRNA), telomere-specific small RNAs (tel-sRNAs), tRNA-derived small RNA, Pyknons, tRFs, and tiRNAs (Table 1). The pervasive transcription detected in mammalian genomes, together with the numerous processes leading to the regulation of ncRNA expression, posit a universe of ncRNA in which their specific biological roles are yet to be explored [7,8].

Among all ncRNAs, tRNAs play essential roles in transporting amino acids into the ribosomes to facilitate mRNA coordinated protein biosynthesis. tRNAs are a group of RNAs well-known for their cloverleaf secondary structure and L-shaped tertiary structure. Specific enzymes, exonucleases, can selectively break tRNAs into several small fragments during biogenesis. This cleavage results in two major classes of tRNA-derived fragments tRFs and tRNA halves (tsRNAs) (Table 1). The biological importance of these short RNAs is yet to be determined [18]. tRNA cleavage products exert similar effects to miRNA [19]; for instance, tRNA^GlyGCC^ showed functional similarities to miRNAs in B-cells lymphoma, specifically interacting with Argonaute (AGO) proteins and downregulating mRNA transcription [20]. Different tRNA fragments, including tRNA-derived stress-induced RNA (tiRNA), have been recently associated with multiple cellular and molecular mechanisms associated with stress responses and diseases such as immune and metabolic disorders, and cancer [18,21,22,23]. This highlights the relevance of deciphering their biogenesis, the effect of exonucleases on their production, and their biological role.

## 2. tiRNA Classification, Biogenesis, and Subcellular Localisation

**Figure 1:**
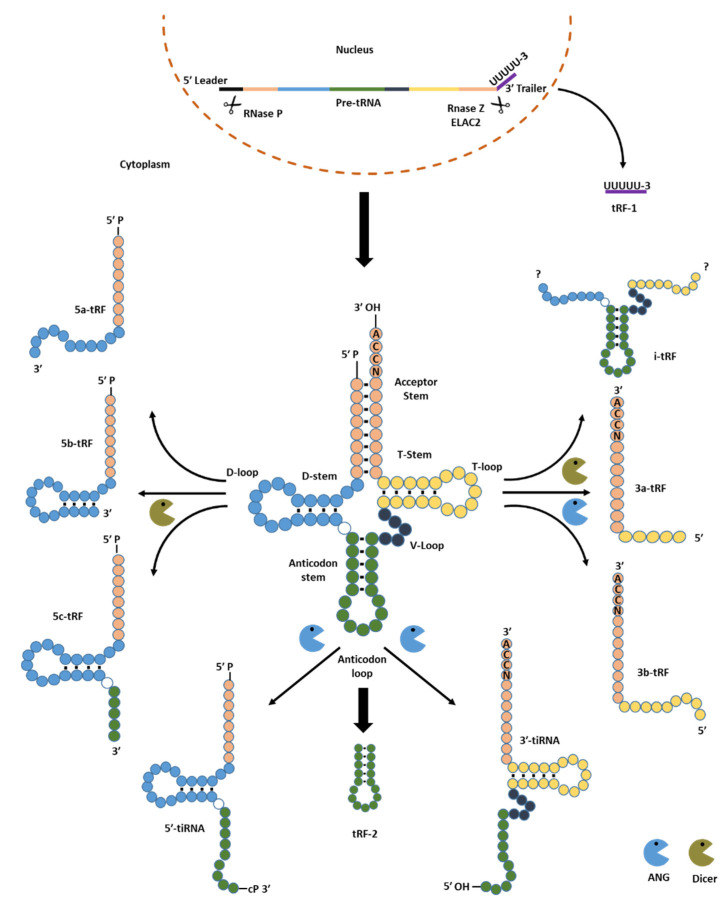
Classification of tRNA-derived fragments (tsRNAs) into tRF and tiRNA (tRNA halves). Graphical representation describing the exonuclease activity of angiogenin and Dicer to digest mature tRNAs.

During their entire lifecycle, tRNAs go through substantial processing that involves a variety of chemical changes [24]. RNA polymerase activity transcribes tRNAs into precursor tRNAs (pre-tRNAs) [25]. Then, the nuclear localised endonucleases Z (RNase Z) together with RNase P cleave the 3′-trailer and 5′-leader sequences from pre-tRNA during tRNA maturation, releasing a 1-tRF characterised by a polyuridine tail [26]. Subsequently, the mature tRNA can be precisely cleaved, mainly by angiogenin, but also from other specific riboexonucleases such as Dicer, generating different structural types (Figure 1). While Dicer is a cytosolic protein, angiogenin is a nuclear protein that translocates and accumulates in the cytoplasm.

Based on the relationship to their parental tRNA sequence, these structural types are classified into six groups as follows. Groups (i) 5-tRF and (ii) 3-tRF, are 14–30 nt in length, and they derive from mature or precursor tRNAs. Groups (iii) 5′-tiRNA commonly known as 5′-tRNA halves and (iv) 3′-tiRNA well-known as 3′-tRNA halves, are 29–50 nt in length, and their generation is induced by stress. The group (v) i-tRFs are ~36 nt in length, it derives from internal parts of tRNAs. Finally, the group (vi) tRF-2, recently discovered tRFs, which carry anticodon stem and loop regions of mature tRNAs [27,28] (Figure 1). It has already been documented that groups i to iv are mainly processed by angiogenin and Dicer [29].

Importantly, Telonis et al. highlighted that the length of various tsRNAs may be tissue-type and tissue-state specific [30]. Moreover, previous studies observed that the distribution of length of tsRNAs revealed that tRNAs with a high percentage of G:C pairs in the T stem produce, by far, more 5′-tRNA fragments than 3′-tRNA fragments. Conversely, tRNAs with 100% G:C pairs in the D stem mainly produced 3′-tRNA fragments. This suggests that tRNA fragment abundance is related to its sequence G:C composition and secondary structure [31]. Further studies showed that the stability and the abundance of certain tRNA fragments can be impacted by sequence modifications [32,33].

In 1990, Levitz et al. [34] reported for the first time, the cleavage of tRNA upon phage-T4 infection. The first fundamental evidence indicating the regulatory properties of tRNAs on human urinary bladder carcinoma was reported about 10 years later [35]. Ten years later, Yamasaki et al. and Fu et al. discovered the role of angiogenin on tRNAs fragment generation [36,37]. Initially, it was thought that most of tsRNAs were found in the cytoplasm [38], where exonucleases such as Dicer and angiogenin can specifically cleave the tRNAs into tRFs [34] and tiRNAs [39]. However, tsRNAs have been recently detected in the serum of small extracellular vesicles of humans, rats, and mice. Notably, mice and rats display a large abundance of tRNA-derived noncoding RNAs as well as miRNAs [40]. Similarly, tRNA-encoding genes have been found in mitochondria across different taxa (*n* = 22) [41]. Specific cleavage of these mt-tRNAs generates mt-tsRNAs that differ in sequence and length from nuclear tsRNAs [42]. Although the cleavage and transport of the mt-tRNAs remain yet to be experimentally validated, two mechanisms have been proposed. In the first one, mt-tRNAs are transported out of the mitochondria into the cytoplasm, and subsequently processed by Dicer and AGO2 proteins. In the second one, mitochondrial localised Dicer and AGO2 process the mt-tRNAs into mt-tsRNAs, and then these are transported into the cytoplasm. Once in the cytoplasm, these mt-tRNAs may take part in the regulation of nuclear-encoded genes [43,44]. The mt-tsRNAs have been recognised in cardiac tissue, where they play a crucial function in mitochondrial protein translation [28]. Importantly, non-mt-tRNAs can also be imported into the mitochondria, which suggests they may also play important roles in mitochondrial gene regulation [45].

## 3. Regulation of tiRNA Processing by Angiogenin

Angiogenin gene (*ANG*), also known as RNase5, encodes for a 14.4 kDa protein that is specific to vertebrates (Table 2). This gene acts as a neovascularisation enhancer [46] and it is also involved in pathologies such as cancer and neurodegeneration [47,48].

The specific cleavage activity on tRNAs suggests that angiogenin-induced tRNA cleavage occurs during stressful events such as starvation, hypoxia, and hypothermia [36,37]. Similar conserved mechanisms determining cell survival and damage [53] have been reported in *S. cerevisiae*, where yeast protein (*Rny1p*), an RNase component of a different family than angiogenin, generates tRNA cleavage upon oxidative stress. Under homeostatic conditions, angiogenin is retained in the nucleus, where it is held in a latent form by its direct inhibitor RNH1 (RNase inhibitor 1) [54]. Upon stress stimuli, angiogenin dissociates from RHN1, probably caused by post-transcriptional modifications mainly asserted by protein kinase C (PKC and cyclin-dependent kinases (CDKs), which phosphorylate angiogenin serine residues and reduce the affinity for its inhibitor, enabling angiogenin–exonuclease activity and promoting tRNAs’ cleavage [55]. RNH1 suppresses angiogenin activity by binding to its catalytic domain of Lys-40, His-13, and His-11 [55]. While *ANG* expression is mainly carried out in the liver, *RNH1* is expressed in endothelial cells as well as in neurons, glial cells, erythroid cells, and osteoblasts, among other cell types.

An important regulator of *ANG* expression is the transcription factor (TF) HIF-1α siRNA-mediated knockdown of the *HIF-1*α gene revealed that HIF-1α is likely to bind the promoter region of the *ANG* gene and downregulate its expression [56]. Similar studies have also shown that *ANG* overexpression in HEK293T and U2OS cell lines results in the upregulation of 5′-tiRNAs (up to 5.7-fold) and 3′-tiRNAs (up to 11-fold) [57], with specifically enhanced production of 5′-tiRNAs of 31–36 nt and 3′-tiRNAs of 36–41 nt in length [57]. Interestingly, overexpression of *ANG* also results in the up-regulation of miRNAs and down-regulation of tRNA-derivated piRNAs (td-piRNAs) expression, showing the involvement of angiogenin in the biogenesis of other sncRNAs [57].

**Figure 2:**
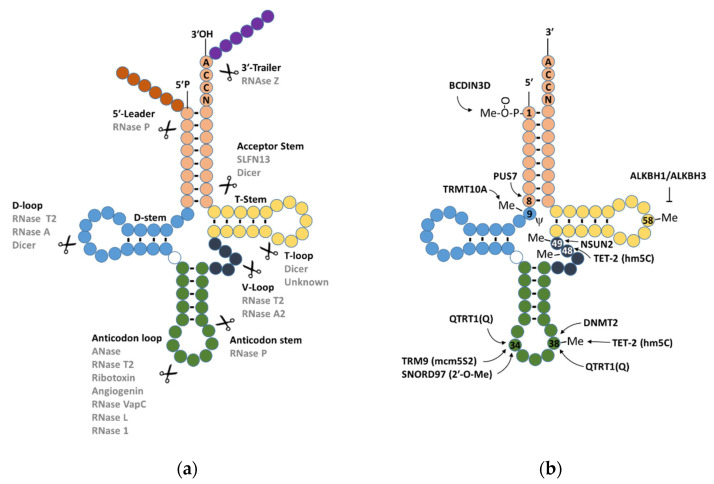
(**a**) Known RNases involved in tsRNA biosynthesis. (**b**) Known enzymes involved in tRNA modification.

Specific types of tRNAs, such as tRNA^Glu^, tRNA^Gly^, tRNA^Lys^, tRNA^Val^, tRNA^His^, tRNA^Asp^, and tRNA^SeC^, are more sensitive to cleavage by angiogenin activity [37,57,58]. The preference for angiogenin cleavage seems to be associated with either the selectivity of riboexonuclease to certain substrates, [57,59] or increased stability of specificity for tRNA species [60]. While the selectivity may be influenced by sequence and tRNA modifications [59,61] (Figure 2b), 5′-tiRNA derived from tRNA^Gly^ and tRNA^Glu^ revealed dimerization processes that may confer higher stability [60]. CRISPR-Cas9-mediated Knock-out of the *ANG* gene in HEK293T and U2OS cell lines showed that only specific tiRNA are affected, whereas most of the tiRNAs were produced via angiogenin-independent processes [57]. This suggests that aside from angiogenin, there are other RNases able to process the cleavage of tiRNAs. For instance, RNase A, a ribonuclease from vertebrates, despite the stress-induced cleavage of tRNAs, is a highly conserved mechanism in bacteria and plants [53,62,63]. Further evidence of independent angiogenin cleavage mechanisms is provided by several studies describing other RNases’ activities [64], such as RNase L [65], RNase 1 [66], Schlafen13/SFLN13 [67], and Dicer [68] (Figure 2a).

## 4. Role of tiRNA in Development, Cell Differentiation, and Apoptosis

**Figure 3:**
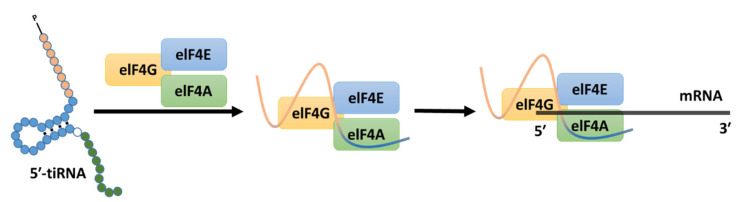
Cleavage of specific tRNAs in 5′-tiRNAs can bind the G4 complex, displacing the elF4F factor and consecutively suppressing the translation by creating an RNA G-quadruplex (RG4s) on the mRNA cap that inhibits translation initiation complex.

While there is abundant information about the role of specific subsets of sncRNAs, such as miRNAs, piRNAs, and siRNAs, in development and cell differentiation, the role of tiRNAs is still poorly understood [69]. Previous studies have shown evidence of the regulatory roles of sncRNAs during cell differentiation. During development, sncRNAs are also expressed in tissue and cell-specific ways suggesting that they play a role in protein expression by interacting with promoter regions and modulating gene expression, for instance, in neurons, muscle, and germ cells [70]. Previous studies showed that several instances of differential tRNA gene expression resulted in changes in the abundance of tRNA-derived fragments but not of mature tRNAs. This shows that noncanonical tRNA activities can be modulated by selectively expressing tRNA genes without harming the mature tRNA pool. This process may be critical for the control of tiRNA functions at the tissue level [71]. It has also been demonstrated that specific sets of 5′-tiRNAs, such as derived from tRNA^GlyGCC^, tRNA^ValCAC^, tRNA^GlnCTG^, tRNA^GluTTC^, and tRNA^LysTTT^, have an important roles during development [69]. These specific sets of 5′-tiRNAs are able to interact with a selected group of proteins and transcripts during mouse embryonic stem cells (mESCs) differentiation. For example, they interact with Igf2bp1, an RNA-binding protein that regulates the expression and translation of c-Myc, an important TF proto-oncogene in cell differentiation and transformation [69,72]. Further studies on hematopoietic stem cells identified the role of the Pseudouridine Synthase 7 (PUS7) on protein synthesis and cell growth. PUS7 mediates the pseudouridylation and it is an important enzyme for epigenetic modification [73]. Downregulation of *PUS7* disrupts tRNA-derived fragment-mediated translation control in embryonic stem cells, specifically 5′-tiRNAs derived from tRNAs harbouring a 5′ terminal oligoguanine (TOG), such as tRNA^Ala^, tRNA^Cys^, or tRNA^Val^. This disruption results in increased protein production and faulty germ layer determination [74]. Moreover, 5′-tiRNA^Ala^ and 5′-tiRNA^Cys^ harbouring a terminal TOG motif, inhibit translation by establishing an intermolecular RNA G-quadruplexes (RG4), replacing the translational initiation complex eIF4G/eIF4E on the mRNA cap (m7GTP) structure [18,75,76] (Figure 3).

tiRNAs seem to also play important roles in aging and spermatogenesis. The aging process comprises several physiological processes that have been summarised in eight hallmarks: loss of proteostasis, stem cell exhaustion, altered intercellular communication, deregulated nutrient sensing, cellular senescence, telomere attrition, mitochondrial dysfunction, genomic instability, and epigenetic alteration [77]. According to studies in human and model species, tiRNAs, and more generally tsRNAs, exhibit diverse expression patterns in the settings of age-related illnesses and physiological senescence.

It has been observed that the composition quantity of circulating 5′-tiRNAs changes during the normal aging process, with a decrease mainly in tRNA^CysGCA^ and tRNA^LysCTT^-derived 5′-tiRNAs, and an increase in tRNA^HisGTG^ and tRNA^AspGTC^ in mice [78]. Other tiRNAs have also been detected as differentially expressed during aging (Table 3), which suggests their putative role in the process.

Of note, 5′-tiRNAs are the predominant tsRNA fragments in motile spermatozoa of several mammals, raising the hypothesis of an active role during late-stage spermatogenesis and zygotic programming [84,85,86]. The high concentration of 5′-tiRNA in mammalian motile sperm is regulated by tRNA methyltransferase 2 (DNMT2), which primarily catalyses the methylation of tRNAs to yield 5′-methylcytosine (m5C) modification [33,87]. Similarly, 3′-tiRNAs have also been identified in human sperm, but are not as abundant as 5′-tiRNAs [84,88]. In addition, the interaction of tiRNAs with PIWI, a family of proteins involved in germ cell maintenance and gamete differentiation [89], suggests their important role in mammalian reproduction and development. For instance, tRNA^ValCAC^ halves retrieved in gonads and kidneys of non-stressed animals interact with the PIWIL4 protein, into a PIWIL4-piRNA complex, which putatively works as translation initiation and efficiency factors [90].

Apoptosis is another key developmental process controlled by specific gene expression, where two signalling cascades come together to activate a set of effector caspases, caspase 3 and 7. The activation of the apoptotic process leads to the release of Cyt-C, which associates with Apaf-1 and activates the two effector caspases resulting in apoptotic degradation [91]. tiRNAs interaction with Cyt-C to form the RNPs complex has also been associated with the regulation of apoptosis during osmotic stress [92]. Therefore, these ANG-derived tiRNAs are key for inhibiting the formation of the apoptosome [92].

## 5. Role of tiRNA on Immunity and Their Potential as Cross-Species Biomarkers

tiRNAs have been recognised as potential systemic immune signalling molecules [93]. 5′-tiRNAs circulate in the bloodstream and may work as extracellular miRNAs in response to viral infections and immune reactions [94]. These tiRNAs may induce the independent assembly of stress-related granules, translating messenger ribonucleoproteins (mRNPs) that can temporarily silence mRNAs, thus helping cells to survive. However, unlike circulating miRNAs released in all peripheral tissues, circulating 5′-tiRNAs appear to be localised mainly in haematological and lymphoid tissues, thus suggesting their function in the immune system [80].

**Figure 4:**
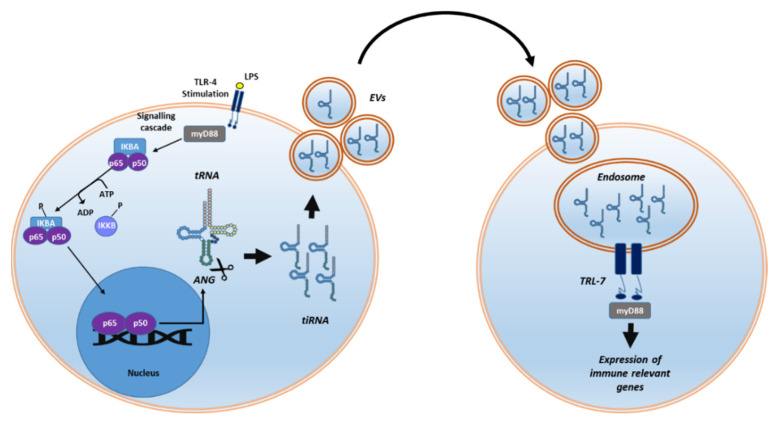
The stimulation of the membrane by TLR activates NF-kB and leads to overexpression of *ANG*. Angiogenin cleaves the anticodon loops of tRNAs producing 5′-tiRNAs that are secreted as signalling molecules after being packed into EVs. TLR-7 is activated when the complex EV-5′-tiRNAs are transported into endosomes in targeted cells, followed by the activation of immunological responses.

The roles of tiRNAs in relevant pathways such as NF-κB, IFN, and Wnt/β-Catenin also suggest their role in immune responses [95]. For instance, the activation of Toll-like receptor 4 (TLR-4) with bacterial lipopolysaccharides (LPS) induces a signalling cascade that enhances NF-κB activation and promotes the expression of *ANG* exonuclease. Cytosolic angiogenin mediates the cleavage of 5′-tiRNAs that are released as signalling molecules after being selectively packed into extracellular vesicles (EVs). The complex EV-5′-tiRNA is then transported into endosomes in recipient cells, activating the TLR-7, and enhancing the expression of immune-related genes (Figure 4) [96]. Although the selection mechanism of specific tiRNAs is yet unknown, discriminatory EV integration of miRNAs and tRFs has also been demonstrated [97,98,99].

Interferon expression is regulated by JAK/STAT pathway activation. Upon viral infection, interferons are secreted and reduce the expression of the *ANG* gene [100].

**Figure 5:**
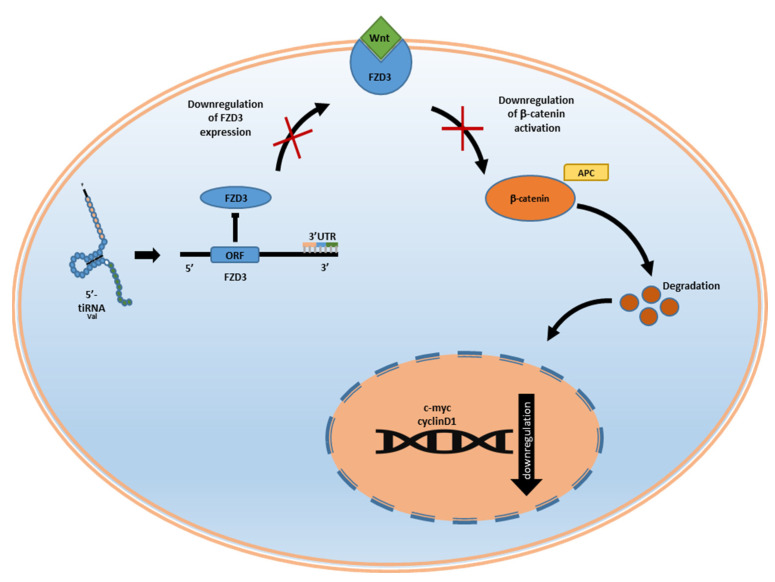
5′-tiRNA^Val^ downregulates the Wnt/β-catenin signalling pathway by targeting *FZD3* gene expression.

Downregulation of *ANG* alters the biogenesis of tiRNAs, changing it into an antiviral state-specific tiRNAs production. The biogenesis of this specific pool of tiRNAs has been proposed to be dependent on other RNases, such as those from the Schlafen family, recently described for their ability to degrade the 3′ end to generate complete 5′ fragments [67]. Another example is 5′-tiRNA^Val^, which is able to directly bind the human Frizzled homolog 3 (*FZD3*) and mediate the downregulation of one of the most critical factors of the Wnt signalling pathway. This mediation controls the formation of effector T cells, the activation of regulatory T cells, and the maturation of dendritic cells (Figure 5) [81,101]. In addition, the genomic localisation of the largest tRNA cluster in humans and other mammals, suggests an important relationship between the immune system and tiRNAs. In humans, the largest tRNA gene cluster is localised in the major histocompatibility complex (MHC) [102].

Bearing in mind that tiRNAs fragments have a role in a variety of gene regulatory mechanisms and influence pathways that are linked to animal features that are economically relevant, they represent possible biomarkers of relevance for detecting and understanding infectious illnesses in farm animal species [103]. In the last few years, the characterisation of sncRNAs in food for humans has triggered several studies in the field of nutrigenomics. For instance, milk includes tiRNAs and tRFs that are encapsulated inside EVs, making them resistant to digestion and potentially absorbable by humans [104]. Because of their great conservation, tsRNAs might be used to mediate cross-species gene expression control, which could have a wide spectrum of uses in the context of human nutrition. Meat quality is affected by fat content, which is regulated by several genes such as *LATS2,* which regulates fat metabolism [105]. A specific tRNA half, 5′-tiRNA^HisGTG^, regulates the expression of *LATS2* [106], which may also associate with meat quality. Additionally, differences in tRNA expression profiles have been identified in large offspring syndrome (LOS) in ruminants, which has been identified as a consequence of assisted reproductive technologies (ART) such as in vitro fertilisation and embryo transfers [31]. There are several tiRNA involved in farm animal diseases that may represent a risk for human health as well (Table 4). Taken together, regulation of the sncRNA milk profile by nutrition, both in humans and dairy cattle, livestock rearing practices, feeding technology, as well as the development of new-born formulae are just some examples where the use of tRNA fragments (tiRNAs and tRFs) as biomarkers may have a great impact in animal and human health [107].

## 6. Role of tiRNAs in Stress Signalling and Behaviour

**Figure 6:**
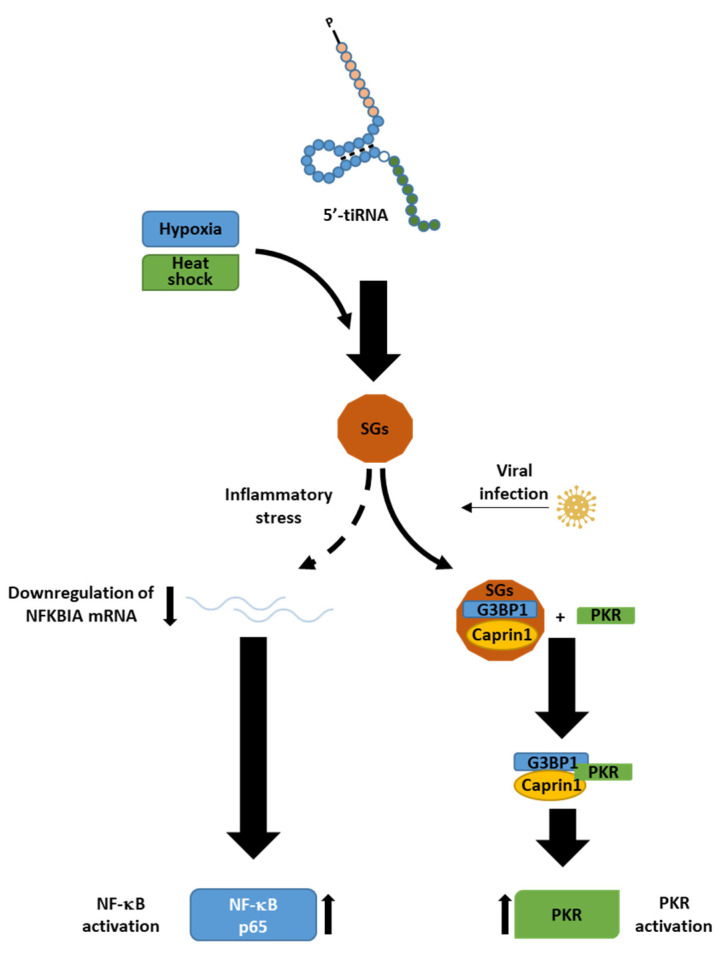
Stress granule formation is mediated by tiRNAs. 5’-tiRNAs promote the formation of stress granules (SGs), phase-separated cytoplasmic ribonucleoproteins assemblies lacking in membranes that condense mRNAs, ribosomal components, RNA binding proteins, and translation initiation factors [114], in response to stress conditions such as hypoxia and heat shock. Through the activation of NF-κB and PKR, SGs play a role in immunity. SGs reduce the production of NFKBIA mRNA, which would normally block the NF-κB pathway during the inflammatory process. As a result, SGs boost the NF-κB pathway. Virus infection also increases the association of G3BP1, Caprin1, and PKR, resulting in the G3BP1-Caprin1-PKR complex. SGs play a role in PKR activation through the G3BP1-Caprin1-PKR complex.

Stress signalling is a cellular communication system that increases cellular stress response. Stress signalling can either minimise potential harm or cause apoptosis depending on the intensity of the exposure. The c-Jun N-terminal kinases (JNK) and p38 MAPK signalling pathways regulate adaptive responses to intracellular and extracellular stressors, including UV radiation, heat, and hyperosmotic circumstances, as well as inflammatory cytokines stimulation [113]. Stressed cells enhance the expression of *ANG*, and its translocation into the cytoplasmic compartment, where it cleaves tRNAs at the anticodon loop to generate tiRNAs and promote stress granule biogenesis (Figure 6) [37].

Previous studies have revealed putative regulatory functions of tiRNAs in porcine brain [115]. While expression profiles differences between the limbic and the hypothalamic–pituitary–adrenal (HPA)-axis tissues suggested that the limbic brain (amygdala and hippocampus) displays a remarkably large concentration of 3’-tiRNAs in phenotypes associated with copying behaviour in pigs, the adrenal gland, an important tissue for regulating the HPA-axis responses to stress, lacked unique 3′-/5′-tiRNA profiles [115]. Similarly, tRFs were found exclusively in the brain tissues, and 3′-tiRNAs showed enrichment in the amygdalar–hippocampal complex [115]. Whereas, 5′-tiRNAs were equally expressed in all four tissues (amygdala, hippocampus, adrenal gland, and hypothalamus) [115]. This suggests that tRNA-derived fragments (tiRNAs and tRFs) show a tissue-specific distribution [115].

Specific sets of tiRNAs may be connected to the coping behaviour in pigs. Upon early life stress, the amygdala from a low reactive haplogroup displayed 5′-tiRNA and 5′-tRF (5’-tRF^Lys^, 5’-tiRNA^Lys^, 5’-tiRNA^Cys^, and 5’-tiRNA^Gln^) enrichments in pigs. Whereas the hypothalamus from the high-reactive haplogroup evidenced an enrichment of 3′-tiRNAs, including 3′-tiRNA^Gln^, 3′-tiRNA^Asn^, 3′-tiRNA^Val^, 3′-tiRNA^Cys^, and 3′-tiRNA^Ile^. The same study suggests that 5′-tiRNA^Leu^, together with other miRNAs and mRNAs molecules, may have a specific function as a gene regulator and signalling molecules across coping behaviour haplogroups [116]. Comparative transcriptomic studies between primates and other mammals also revealed different expression profiles across species, suggesting that 5′-tiRNAs are more expressed in the hippocampus in primates, pigs, rats, and mice. 5′-tiRNA^GluCTC^ and 5′-tiRNA^GlyGCC^ were the most abundant among all the 5′-tiRNA, which suggest a well-conserved role in primate’s hippocampus neurogenesis [117].

## 7. tsRNA Databases

**Figure 7:**
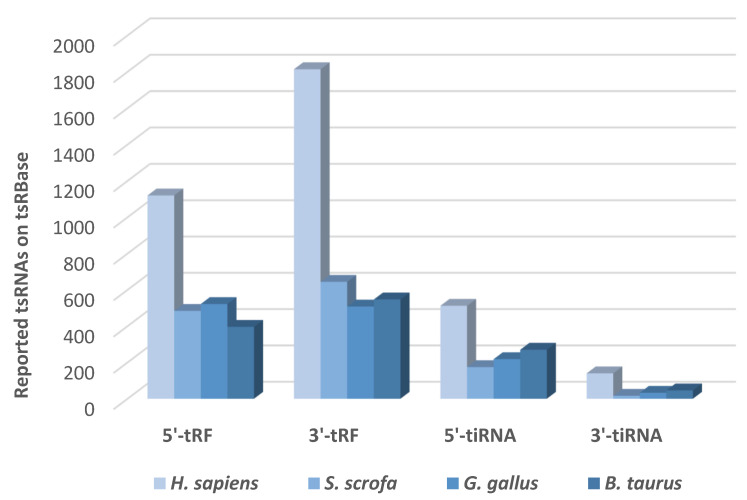
Reported tsRNAs in *Homo sapiens*, *Sus scrofa*, *Gallus gallus,* and *Bos taurus* on tsRBase.

The expansion of RNA high-throughput sequencing techniques has improved the continuous discovery of tsRNAs. Various tsRNAs databases have been created to facilitate the access and study of their molecular roles in different physiological processes (Table 4) [118]. tRFdb was the first database of transfer RNA-related fragments. It collects official names, read counts, and sequences of tRNA fragments from eight organisms: *R. sphaeroides*, *S. pombe*, *D. melanogaster*, *C. elegans*, *X. tropicalis*, *D. rerio*, *M. musculus,* and *H. sapiens* [119]. The tRFexplorer database allows users to visualise the expression profiles of tRNA-derived ncRNAs in each NCI-60 cell line panel. This enabled the assessment of tRNA-derived ncRNA expression with CellMiner Omics and chemical activity data. Such a step represents a good opportunity to examine their putative biological significance in the lack of experimental validation [120]. The tsRBase database contains tsRNA IDs, sequences, source tRNAs, and expression values for approximately 14,000 samples from 20 different species, including vertebrate and invertebrate, plants, bacteria, and fungi (Figure 7) [121]. Another interactive database is GtRNAdb, a tool that allows exploring gene predictions. GtRNAdb is continuously increasing content wise, and it is the most often cited web-based repository of tRNA gene information (Table 5) [122]. In comparison to tRFdb ID and MINTbase ID, tDRnamer offers a consistent, stable name and extra annotation for each submitted tDR sequence [103]. Other tools, such as tRNA Analysis of eXpression (tRAX) allows the analysis of tRNA-derived small RNAs (tDRs) and mature tRNAs, and the inference of RNA changes from high-throughput small RNA sequencing data [104] (Table 6).

## 8. Conclusions and Future Perspectives

Since the earliest reports on the non-ribosomal functions of tRNAs, new data have revealed the “non-canonical” functions of tRNAs. The list keeps growing, especially with the expansion of the types and the description of the cellular-specific features of tRNA fragments. These types and features have considerably attracted attention in the last 5 years in areas such as human health and disease, and, also, in physiological responses to stress-related environmental factors. In this review, we describe the physiological mechanisms by which tiRNAs are generated and how they take part in multiple essential physiological regulatory mechanisms and pathways. We also present a collection of useful databases of great use for further exploring tiRNAs.

Despite the emerging interest and the constantly increasing information, many aspects of tiRNAs and generally about tRNA fragments are not clear yet. Unfortunately, tRFs and tiRNA have been lately rediscovered from small non-coding RNA data sets. This was probably due to experimental designs mainly focused on miRNAs where sequences longer than 18–24 nt were routinely deleted, and reads that aligned with tRNAs, rRNAs, or snoRNAs were deemed as degradation products, thus being removed during the quality controls [123].

The new discovery of novel ncRNAs has expanded our knowledge about their tissue-specific expression patterns, diverse interactions, and subcellular locations, something that will surely expand our comprehension of their putative functional regulatory activities, among other physiological roles.

As potential biomarkers, tsRNAs have promising value in the fields of human and animal health. Similar to miRNAs biomarkers [124], tsRNAs may offer additional measures of biological states for stress associated with anthropogenic, environmental (including temperature stress), pathogen, and aging-related factors in humans, but also in farm animal research, as already shown in pigs [125]. The composition, stability, and abundance of tRFs and tiRNAs are highly dependent on cell type and disease state and are highly enriched in biofluids, much more so than microRNAs, making them excellent biomolecules for biomarkers. It is plausible to use the level of tsRNAs as a potential biomarker, by comparing it with conventional biomarkers such as cortisol level. The validation of results in a large number of animals for potential uses as molecular biomarkers in determining the health status of farm animals is necessary.

## Figures and Tables

**Table 1:** Mammalian non-coding RNA classes.

	Class	Symbol	Characteristics	Biological Functions Associations	Ref.
***Small non-coding RNAs (sncRNAs)***	**MicroRNAs**	miRNAs	18–26 nt; comprises 2% of human genome and regulate up to 50% of protein-coding genes.	Control of proliferation, apoptosis, and differentiation.	[9]
	**Small interfering RNAs**	siRNA	19–23 nt; processed by Dicer, and guide sequence-specific degradation of target mRNA.	Post-transcriptional regulation of gene expression.	[10]
	**Piwi-interacting RNAs**	piRNAs	24–31 nt; made by single-stranded RNA (ssRNA) precursors and it is Dicer-independent.	Embryonic development, germline DNA integrity, transposon transcription silencing, translation suppression, heterochromatin creation, and sex determination epigenetic control.	[11]
	**Small nucleolar RNAs**	snoRNAs	60–300 nt; divided into two classes: C/D box snoRNAs and H/ACA box snoRNAs. It is primarily accumulated in the nucleoli.	Responsible for post-transcriptional modification and maturation of ribosomal RNAs (rRNAs), small nuclear RNAs (snRNAs), and other RNAs (snRNAs).	[12]
	**Centromere repeat-associated small interacting RNAs**	crasiRNAs	34–42 nt; processed from long dsRNAs.	Activation of heterochromatin and centromeric proteins.	[13]
	**Telomere-specific small RNAs**	tel-sRNAs	~24 nt; pi-like small RNA and independent of Dicer processing.	Epigenetic regulation.	[14]
	**Pyknons**		>16 nt long; observed in groups in intergenic and intronic domains.	Primarily engaged in cell communication, transcriptional regulation, signalling, and transport.	[15]
	**tRNA fragments**	tRFs	14–30 nt; dependent on angiogenin and Dicer processing.	Diverse molecular and physiological processes, including gene suppression, RNA processing, protein translation, stress responses, cell proliferation, and differentiation.	[16]
	**tRNA-derived stress-induced RNAs**	tiRNAs (tRNA halves)	29–50 nt; the most abundant right downstream of transcriptional end sites. It exhibits spatial preservation patterns and predominantly resides in GC-rich promoters.	Control of protein-coding gene transcription by targeting epigenetic silencing complexes.	[17]

**Table 2:** *ANG* and pseudogenes discovered in mammals and higher vertebrates.

*Species*	*ANG*	Protein Length	Ref.
**Human *(H. sapiens)***	1	147 aa	[49]
**Mouse *(M. musculus)***	5 (3 pseudogenes)	145 aa	[49]
**Rat *(R. norvegicus)***	2	145 aa	[49]
**Dog *(C. lupus familiaris)***	1	145 aa	[50]
**Cattle *(B. Taurus)***	3	148 aa	[50]
**Pig *(S. scrofa)***	2	202 aa	[51]
**Rabbit *(O. cuniculus)***	1	149 aa	[51]
**Rainbow trout *(O. mykiss)***	6	205 aa	[52]
**Zebrafish *(D. rerio)***	5	149 aa	[52]

**Table 3:** tiRNAs linked to aging hallmarks and aging-related diseases.

Aging Hallmarks	tiRNA ID	tiRNA Type	Gene or Protein Target	Mechanism	Ref.
***Cellular senescence***	tiRNA-5034-GluTTC-2	5′-tiRNA	-	Downregulated in cancer tissue, and the degree of expression is inversely related to tumour growth.	[79]
	tiRNA^Lys^	5′-tiRNA	-	Regulation of aging hallmarks	[78]
***Loss of proteostasis***	tiRNA^Ala^	5′-tiRNA	elF2a	Inhibits protein synthesis	[80]
	tiRNA^Cys^	5′-tiRNA	elF2a	Inhibits protein synthesis	[80]
	tiRNA^Val^	5′-tiRNA	*FZD3*	Regulation of WNT/β-Catenin pathway	[81]
***Aging related diseases***	tiRNA^AsnGTT^ tiRNA^IleAAT^ tiRNA^AspGTC^	5′-tiRNA	-	Downregulation observed in rheumatoid arthritis patients	[82]
	tRNA^Val^ derived	5′-tiRNA 3′-tiRNA	Aβ/Tau	May inhibit Aβ production and Tau protein hyperphosphorylation in Alzheimer’s disease	[83]

**Table 4:** tiRNAs involved in some human and farm animal infectious diseases.

*Disease*	tiRNA ID	tiRNA Type	Mechanism	Ref.
*Bovine viral diarrhoea virus (**BVDV**)*	tiRNa^GlyCCC^ tiRNA^GlyGCC^	5′-tiRNA	It is downregulated, it may define the immune response against BVDV	[108]
*Respiratory syncytial virus (**RSV**)*	tiRNA^GluCTC^ tiRNA^Glu^	5′-tiRNA	RSV promotes *ANG* expression, and then cleaves specific tRNAs that may inhibit *APOER2* production	[109]
*Bovine Leukemia* *Virus (**BLV**)*	tiRNA^GlnCTG^ tiRNA^GlnTTG^ tiRNA^HisGTG^	5′-tiRNA	It targets white blood cells causing dysregulated immune functions and immunosuppression.	[110]
*Mycoplasma bovis*	tiRNA^SelCysUGA^	5′-tiRNA	It may be correlated with a host defence mechanism enhanced by bacterial infection.	[111]
*Trypanosoma brucei (**rHAT**)*	tiRNA^Thr^	3′-tiRNA	During the stress recovery phase, it attaches to the ribosome and increases protein production.	[112]

**Table 5:** tRNA Gene Summary of *Homo sapiens*, *Sus scrofa*, *Gallus gallus,* and *Bos taurus* on GtRNAdb v2 (accessed on 9 April 2022).

Organism	tRNA Decoding Standard 20AA	TCA Suppressor tRNAs	Genome Version
*H. sapiens*	415	1	Feb.2009 GRCh37/hg19
*S. scrofa*	471	1	Feb.2017 Sscrofa11.1
*G. gallus*	280	1	Mar.2018 GRCg6a
*B. taurus*	619	1	Apr.2018 ARS-UCD1.2/bosTau9

**Table 6:** tiRNAs and tFRs databases including mammals and other vertebrates.

*Name*	Description	Link (accessed on 9 April 2022)
*tRFexplorer*	Publicly accessible database that allows users to view the expression profiles of tRNA-derived ncRNAs in each NCI-60 cell line.	https://trfexplorer.cloud/
*tRFdb*	The first database of transfer RNA fragments (tRFs)	http://genome.bioch.virginia.edu/trfdb/
*tsRBase*	Multi-species database of tsRNA sequences, expression characteristics, and function.	http://www.tsrbase.org/
*GtRNAdb v2*	tRNA gene predictions on complete or nearly complete genomes.	http://gtrnadb.ucsc.edu/GtRNAdb2/
*tDRnamer*	Standardised naming for tRNA-derived RNAs	http://trna.ucsc.edu/tDRnamer/
*tRAX*	In-depth analysis of tRNA-derived small RNAs (tDRs), mature tRNAs, and RNA modification inference from high-throughput small RNA sequencing data.	http://trna.ucsc.edu/tRAX
